# Beyond clinical food prescriptions and mobile markets: parent views on the role of a healthcare institution in increasing healthy eating in food insecure families

**DOI:** 10.1186/s12937-020-00616-x

**Published:** 2020-09-09

**Authors:** Emily L. DeWit, Emily M. Meissen-Sebelius, Robin P. Shook, Kimberly A. Pina, Evelyn Donis De Miranda, Michelle J. Summar, Emily A. Hurley

**Affiliations:** 1 Center for Children’s Healthy Lifestyles and Nutrition, Children’s Mercy Kansas City, Kansas City, MO USA; 2 Health Services and Health Outcomes Research, Children’s Mercy Kansas City, Kansas City, MO USA; 3 Department of Pediatrics, University of Missouri Kansas City- School of Medicine, Kansas City, MO USA

**Keywords:** Mobile market, Food prescription, Food insecurity, Pediatric primary care, Fruit and vegetable consumption, Food access

## Abstract

**Background:**

Children in food-insecure families face increased barriers to meeting recommendations for fruit and vegetable consumption. Hospitals and pediatric healthcare institutions have attempted to alleviate food-insecurity through various internal programs like food prescriptions, yet little evidence for these programs exist. Consistent with a patient-centered perspective, we sought to develop a comprehensive understanding of barriers to fruit and vegetable consumption and a parent-driven agenda for healthcare system action.

**Methods:**

We conducted six qualitative focus group discussions (four in English, two in Spanish) with 29 parents and caregivers of patients who had screened positive for food-insecurity during visits to a large pediatric healthcare system in a midwestern U.S. city. Our iterative analysis process consisted of audio-recording, transcribing and coding discussions, aiming to produce a) a conceptual framework of barriers to fruit and vegetable consumption and b) a synthesis of participant programmatic suggestions for their healthcare system.

**Results:**

Participants were 90% female, 38% Black/African American and 41% Hispanic/Latino. Barriers to fruit and vegetable consumption in their families fell into three intersecting themes: *affordability, accessibility* and *desirability.* Participant-generated intervention recommendations were multilevel, suggesting healthcare systems focus not only on clinic and community-based action, but also advocacy for broader policies that alleviate barriers to acquiring healthy foods.

**Conclusion:**

Parents envision an expanded role for healthcare systems in ensuring their children benefit from a healthy diet. Findings offer critical insight on why clinic-driven programs aimed to address healthy eating may have failed and healthcare organizations may more effectively intervene by adopting a multilevel strategy.

## Background

According to the Centers for Disease Control, increasing fruit and vegetable consumption (FVC) among children is a national public health priority [1]. FVC not only provides immediate health and nutritional benefits in childhood [2], but is associated with adult dietary patterns and reductions of chronic diseases, including obesity, cardiovascular disease, type 2 diabetes, and some cancers [3, 4]. National guidelines recommend children consume five or more servings of fruits and vegetables daily, however, recent data suggests only about 10% of children ages 2–18 years meet these guidelines [5].

Children experiencing food insecurity face major barriers to meeting FVC recommendations. Food insecurity is a household-level condition of limited or uncertain access to adequate food, resulting in disrupted eating patterns or reduced food intake [6]. Nationally, about 12% of American households were food insecure for at least some time during 2017, even though most reported participating in one or more nutrition assistance programs (e.g. Supplemental Nutrition Assistance Program (SNAP), Women, Infants, and Children (WIC) & National School Lunch program) [7]. Food-insecure families are two to four times more likely to report barriers to accessing fruits and vegetables [8], and severity of food insecurity has a strong, negative association with fruit and vegetable intake [9].

Consistent with increased efforts to address social determinants of health, hospitals and healthcare systems across the country have started investing in interventions to screen for and address food insecurity among their patients [10, 11]. Patients identified as food-insecure may be linked to services in the community, or to programs initiated within healthcare facilities, such as onsite food pantries, home delivered meals, or food prescriptions [12]. Most of these programs, however, aim to increase general food access, missing the opportunity to target FVC [13–15]. One exception is a fruit and vegetable prescription model, in which health care providers dispense “prescriptions” in the form of coupons or vouchers [16]. However, published evidence on the impact of these prescription programs, particularly within a primary care setting, is lacking.

Our experience suggests that increasing FVC among children in food-insecure families may not be as simple as dispensing food prescriptions. In our pilot program, primary care providers at a large, urban clinic dispensed fruit and vegetable prescriptions to food-insecure families during a clinic visit. The prescription consisted of a $5 voucher, redeemable at a community mobile market that operated from April–October 2017 and exclusively sold low-cost, high-quality fresh produce. During the pilot, 462 coupons were distributed along with a mobile market schedule and educational brochures; however, only 5% of vouchers were redeemed. Following this unsuccessful pilot, we sought to conduct an in-depth examination of barriers and facilitators to fruit and vegetable consumption among food-insecure families.

Consistent with a patient-centered model of care, we also aimed to elicit a parent-driven agenda for interventions pediatric health care institutions can implement to address fruit and vegetable consumption for food-insecure families. Previously, while studies have identified high cost and limited access as barriers [17–19] to fruit and vegetable consumption in food-insecure families, none to our knowledge have sought to generate solutions from the caregiver perspective. Understanding the perspectives from families experiencing food insecurity can help identify opportunities to improve program acceptability, feasibility and effectiveness.

## Methods

### Study setting and participants

We conducted qualitative focus group discussions with parents and caregivers of patients of a primary care clinic embedded in a larger pediatric health care system that serves children from birth through adolescence. According to 2016 data in the hospital’s four-county catchment area, 19.5% of caregivers “often” or “sometimes” worried that their food would run out and 16.0% “often” or “sometimes” ran out of food and did not have money to buy more [20]. We recruited participants who screened positive for food insecurity during their child’s well or sick primary care visits and were given a food prescription during the 2017 pilot program. Participants were considered food insecure if they answered “yes” to one or both of the Hunger Vital Sign™ questions sourced from the Safe Environment for Every Kid (SEEK) questionnaire [21]: “*In the past 12 months,* (1) *did you worry that your food would run out before you could buy more?* (2) *did the food you bought just not last and you didn’t have money to get more?”*

Participants were eligible if they spoke English or Spanish and were at least 18 years old. We forecasted that 5–7 focus groups (6–8 participants each) would be adequate to reach thematic saturation [22], and aimed to conduct 3–5 groups in English and 2 in Spanish. This study was approved by the Institutional Review Board at Children’s Mercy Kansas City.

### Data collection

Focus groups were conducted at various locations within the pediatric facility campus and were led by trained moderators in English or Spanish. A primary moderator led each group with assistant moderator taking notes and recording non-verbal cues such as body language, emphasis and facial expressions [23]. English-speaking focus groups were moderated by the first authors (EMS, ED), while Spanish-speaking groups were led by bilingual/bicultural co-authors (KP, EDD). Moderators and assistants are masters-level research staff with specialized training in focus group moderation. Each discussion was audio-recorded and lasted approximately 90 min. Participants gave verbal consent prior to participation and were offered a $35 gift card, healthy meal, and on-site childcare.

The moderator used a semi-structured guide to lead the discussion. The guide was composed of questions and probes that reflected our aim to identify barriers and facilitators and elicit a parent-driven agenda. We developed the guide after conducting in-person interviews with WIC participants who had received a similar fruit and vegetable prescription in a pilot project. Based on those interviews, as well as phone surveys with patient families who had received the prescription, we reviewed and revised questions for the focus group format. (See detailed information in Focus Group Guide supplement) All authors, as well as physicians from Children’s Mercy’s Hunger Free Hospital Task Force, were involved in developing and reviewing the guide*.* Discussions began with an open exploration of parent’s general concerns regarding food for their family. The discussion continued with questions regarding their experiences getting enough healthy foods (fruits and vegetables) for their family. The latter half consisted of free-listing activities where participants generated their own solutions for what a healthcare institution could do to increase FVC among families. Lastly, ideas were summarized on chart paper, ranked by participants in order of preference and top ideas were discussed further.

### Data analysis

Focus groups were transcribed and uploaded into Dedoose version 8.2.14 [24]. Spanish transcripts were transcribed into English by Spanish-speaking moderators. Analysis methods were informed by grounded theory [25] and began with line-by-line inductive coding of the first English and Spanish transcripts by two independent coders [22]. Preliminary codes were refined through discussion and consensus and organized thematically into a hierarchical codebook. Iterative restructuring of the codebook by group consensus continued as new themes and relations between themes emerged in subsequent transcripts.

Code outputs were reviewed and summarized, and the structure of the codebook became the basis of two final analytic goals: a) a conceptual framework of barriers to FVC and b) a synthesis of participant intervention suggestions, which thematically aligned within a social-ecological framework [26].

Participants suggestions naturally fell into different levels of the socioecological model. As lifestyle behavior change strategies are shifting toward multilevel interventions, it’s useful to examine participants’ ideas within each level of influence [27]. Further, mapping participant suggestions in the socioecological model may be useful for program planning, as healthy lifestyle behavior change programs are increasingly employing multilevel intervention strategies.

## Results

A total of 29 participants attended the six focus group discussions (four in English and two in Spanish). Participants represented various age and racial groups, with the majority [26] female (Table 1).

**Table 1:** Demographics of participants by focus group language

	English-Speaking (4 groups)	Spanish-Speaking (2 groups)	Total
Race/Ethnicity			
Hispanic/Latino American		12	**12** (41.4%)
Black/African American	11		**11** (37.9%)
White/Caucasian	5		**5** (17.2%)
Multiracial	1		**1** (3.5%)
Age			
18–29	3	3	**6** (20.7%)
30–39	7	4	**11** (37.9%)
40–49	1	4	**5** (17.2%)
50–59	4	1	**5** (17.2%)
60 and above	2	0	**2** (6.9%)
Gender			
Female	16	10	**26** (89.7%)
Male	1	2	**3** (10.3%)
**Total**	***N*** **= 17**	***N*** **= 12**	***N*** **= 29**

### Predominant barriers to Fruit and vegetable consumption

**Fig. 1:**
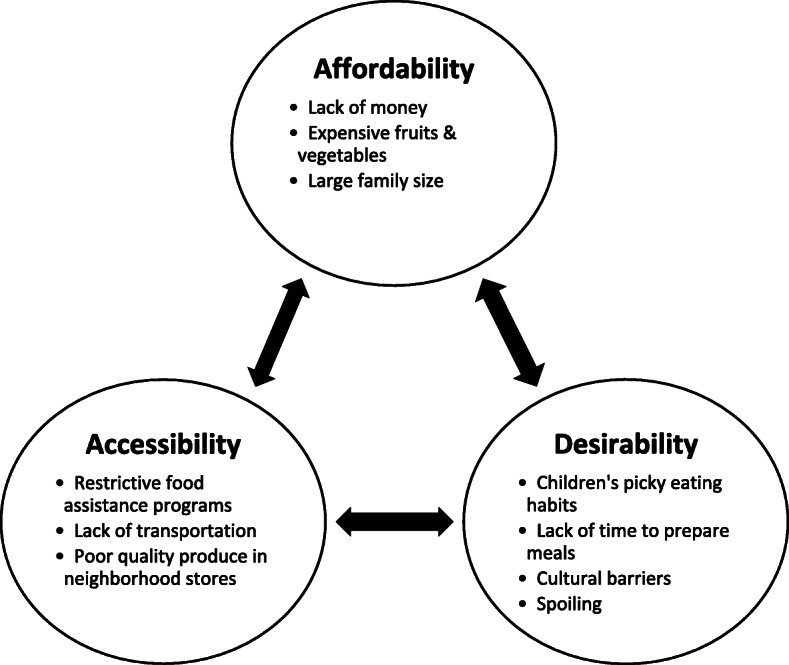
Predominant barriers to fruit and vegetable consumption among food-insecure families

Participant-reported barriers to FVC fell within three interrelated categories: affordability, accessibility, and desirability (Fig. 1).

#### Affordability

Affordability concerns encompassed lack of money to buy food as well as the high price of fruits and vegetables. When asked about their biggest worries regarding food in general, participants’ most salient response was overwhelmingly lack of money.

*“I have $35 a month for food. And that’s like barring the costs of gas going up, or anything going wrong, or needing an oil change for my car”* (English-speaking female, Group 2).

Making sure their children had enough food was a significant stressor, to the extent that some participants reported rationing or forgoing food themselves.

Participant 1: *“But I just don’t eat to make sure that they [my children] are fed.”*

Participant 2: *“I’ve done that*.*”* (commenting in agreement with Participant 1)

When asked specifically about consuming fruits and vegetables, caregivers maintained affordability as the biggest barrier.

*“If parents could find cheaper vegetables, we would be able to buy them*” (Spanish-speaking Female, Group 4).

Participants considered “healthy foods” (fruits and vegetables) more expensive than other foods and could only consider purchasing them after paying for other necessities. As one participant explained:

*“If in this pay period everything has been paid, and I have a little extra […] I let my daughter, pick out, she’ll pick up fruits”* (English-speaking female, Group 2).

*“ I’m on disability and by the time I pay my rent, my lights, my water, my gas and everything is taken care of, what I need, my personal needs, cause I budget, there’s nothing left for me to have in the bank but $25-how do you make it? That’s called knees and pray. You know how you get on your knees and you start praying… And the healthy stuff that you really do need, you really can’t get it”* (English-speaking female, Group 6).

Even within meal planning decisions, participants often regarded fruits and vegetables as an “extra” that they had to forgo in order to afford what they considered more substantial, essential staples.

*“It’s just considering things that are a meal and prioritizing. So, if we have spaghetti, you know that’s our meal and that’s the money... A lot of times, I see vegetables and fruit as extra, or a side, or a snack. So, we can’t get snacks today but we have a meal”* (English-speaking female, Group 2).

Expense of fruits and vegetables was a concern for participants trying the stretch their food budgets to feed large families:

*“Not only is it expensive, when you got 5 kids […] A bag of oranges are gone in a day, an hour”* (English-speaking female, Group 1).

#### Accessibility

Participants also talked about difficulties in accessing affordable, quality fruits and vegetables, including frustration with the lack of transportation and lack of stores selling healthy foods within their neighborhoods.

*“Nothing is close, might have to take several buses to get to the grocery store or buy food at a [gas] station where they mark food up 500%”* (English- speaking female, Group 3).

*“Well, I don’t usually know where to buy [fruits and vegetables] at a lower price”* (Spanish-speaking female, Group 4).

Participants identified accessibility challenges as a major reason for underutilization of the hospital’s pilot food prescription program, as prescriptions could only be redeemed at one mobile market. No participants had actually visited the market or redeemed their $5 “prescription” and only two remembered hearing about the market. They cited inconveniences including the highly variable schedule of the mobile market, the short length of stops (typically 1 h) and the unavailability of non-produce items, requiring parents to make an extra shopping trip. One participant described,

*“It’s so hard to pinpoint where it’s [mobile market] going to be…and then about the time you get to one spot, it has moved to another spot”* (English-speaking female, Group 6).

Participants also identified accessibility barriers to community food assistance programs, including eligibility restrictions for WIC and SNAP (income restrictions, age limits) and limited hours and long lines at food pantries.

Access to quality fruits and vegetables was especially challenging to families relying on food pantries. One participant described the produce at pantries as *“on the edge of expiration”* (English-speaking female, Group 2). While they did not prefer canned goods, several participants saw them as the only option for obtaining fruits and vegetables at food pantries. Families who did not have easy access to pantries faced additional barriers related to affordability and desirability, a choice that one participant explained was impacted by the low quality of pantry food:

*“I can see a pantry desert […] When you look at the economics of it and the price of gas…the time and gas is not worth what the pantries distribute”* (English-speaking female, Group 3).

#### Desirability

Participants also identified several challenges of desirability, or the demand and preference for fruits and vegetables in their families. Participants described desirability barriers including children’s picky eating habits, time and effort required to prepare or cook, as well as cultural traditions.

Many parents wished their children or other adults in the family favored vegetables, as children’s preferences often led to difficulties cooking meals that incorporated fruits and vegetables.

*“When I go on a diet or I try to eat healthier, I have to cook a meal for myself, a meal for my kids, and a meal for my husband. Yes, I have to cook three different meals for them. If we could all eat healthy together, then it would be different”* (Spanish-speaking female, Group 4).

With fruits and vegetables already considered more expensive, low desirability, particularly for vegetables, added an additional deterrent. Parents often saw purchasing fruits and vegetables or trying new dishes as a risk, wasting money on foods their children would not eat. Participants also perceived a high investment of time to purchase and prepare fruits and vegetables, making them less desirable options.

*“When someone goes to the doctor’s visit and they ask if we give our kids vegetables, well we are honest and answer no because it is easier for us to buy McDonald’s, a Happy Meal or something. Because when some parents work, it’s harder for us to cook”* (Spanish-speaking Female, Group 4).

Participants in five of the six groups also described fresh fruits and vegetables as less desirable because of their shorter shelf life.

*“Because it [fruits and vegetables] only lasts so long. You buy it today and bananas be spoiled by tomorrow…”* (English-speaking female, Group 6).

While most of the barriers were consistent across both the English and Spanish groups, many Hispanic parents, in particular, identified struggles to eat healthy when fruits and vegetables are not a typical component of their meals. Hispanic parents, in particular, spoke about cooking consistent with their families’ cultural traditions, preferences and habits, which they described often did not include vegetables.

*“I will make red hot chile with pork meat because my kids like it a lot. And it is easier because you just put a slab of meat and some beans and then they eat. To add vegetables and all that is really expensive”* (Spanish-speaking female, Group 4).

### Participant-generated recommendations for healthcare-based initiatives

Participants believed the healthcare system could have a wide-ranging role in helping children of food-insecure families eat more fruits and vegetables. Major themes included addressing affordability through direct assistance with foods and other basic needs and through advocacy; increasing accessibility through integrating services into their routines while using multiple channels of communication; and promoting desirability through the involvement of families and the influence of clinic providers. Beyond a focus on food, participants discussed other factors that impact a family’s ability to acquire healthy food, such as one Spanish-speaking focus group that emphasized the need to increase access and education surrounding family planning. Table 2 summarizes participants’ intervention ideas across a socio-ecological model [28], and their potential impact to increase affordability, accessibility and/or desirability.

**Table 2:** Participant-generated intervention ideas for healthcare systems to address the barriers of affordability, accessibility and desirability of fruits and vegetables for food-insecure families, by socioecological (SE) level

Barrier(s) Addressed			Intervention Idea	SE Model Level	Description for Healthcare System Action
**Affordability**	**Accessibility**	**Desirability**			
X			Advocacy: Policies that make healthy food more affordable	**Policy**	Advocate for local or national policies to increase affordability of food (e.g. expanding food assistance programs), or overall budget for food (e.g. expanding affordable housing or childcare).
X			Advocacy: Tax-free healthy foods or Tax-free day		Work with local government to establish a tax-free policy for healthy food, or tax-free weekends for healthy food (similar to an existing back-to-school tax-free weekend popular with families in the region).
X	X	X	Accessible mobile markets	**Community**	Create more accessible, convenient mobile markets with low cost fruits and vegetables, or to distribute free food to families
X	X	X	Community gardens		Establish community gardens at schools or healthcare centers. Involve kids and use the garden as a teaching and volunteering opportunity for kids and parents.
		X	Community events		Launch city-wide event to promote healthy eating with family-friendly, hands-on activities. Send doctors to existing community events to promote healthy eating and connect families to resources.
X	X		Healthy food distribution	**Organizational (Healthcare Institutions)**	Distribute free fruits and vegetables through providers at clinic visits and/or healthy eating classes or events.
X			Coupons for healthy food		Provide coupons for fruits and vegetables through providers at clinic visits or with an educational class. Important characteristics of the coupon include no expiration date, ability to use it at multiple locations, and broad eligibility for the coupon (i.e. eligibility would not be income-based, but need-based)
X		X	Office for healthy food		Create an office at the healthcare facility to distribute fruits and vegetables or coupons to families, as well as information about community food resources, nutrition and how to cook healthy, desirable meals.
X			Connect families to community resources		Establish an onsite navigator to connect families with broader community services (e.g. housing, childcare and food assistance programs.) Use a personalized approach with thorough and up-to-date knowledge of resources and eligibility rules by geographic area.
	X	X	Fruit Baskets		Maintain clinic fruit baskets and allow children to pick piece of fruit after their visit.
		X	Workshops/Group Classes	**Family/** **Individual**	Offer hands-on nutrition workshops with emphasis on community partnerships, education and family involvement. Examples included freezer meal and meal preparation classes, and family cooking classes. One Spanish-speaking group also emphasized the need to educate parents on family planning, as large families impact ability to buy food.
		X	Support Groups for Parents		Establish a group where parents can talk, network, and learn from each other about accessing community resources, preparing healthy food and budgeting.
		X	Rewards Program		Create a program for children to earn rewards through the healthcare system for achieving healthy eating metrics.
		X	Educational Handouts		Distribute educational handouts for healthy recipes or substitutions, including material for children with special health care conditions or dietary needs.

### Address affordability

#### Offer direct assistance and linkages to external resources

As affordability was the most significant barrier discussed, parents suggested healthcare institutions offer direct assistance in the form of coupons or onsite food (i.e. bags of fruits and vegetables, onsite pantry). Coupons were most preferred, but participants thought a higher amount, redeemable at more convenient locations would make the program more successful than the pilot food prescription program.

“$*5 would be okay, but if they were to give $20, I would say it’s worth taking and not losing out on it. And I could use it at whatever store I go to, or where I buy the most produce”* (Spanish-speaking female, Group 4).

Participants also recommended that healthcare institutions connect families with existing community resources (affordable housing, affordable childcare, community food resources) to alleviate overall financial constraints.

*“There’s so many different programs, not just food related. Childcare assistance and stuff like that. So many programs out there that I had no clue […] So, I think just being able to give the parents the resources and telling them about the programs”* (English-speaking female, Group 2).

#### Advocate for maintaining or expanding federal and community programs

Participants across groups noted that benefits programs like WIC, SNAP, and school lunches alleviate cost and increase accessibility of fruits and vegetables.

*“If it wasn’t for WIC, then there’d probably be times that we didn’t have any [fruits and vegetables] in our house”* (English-speaking female in group 2).

In addition, parents valued SNAP, especially when extra benefits were offered (extra funds for summer months, or double dollars for farmer’s markets) as well as school food programs:

*“What’s most helpful is making sure she [my daughter] gets to school or is in some type of program that provides food. So that I know that she’s ate […] making sure that she’s at school every day, because I know she’s gonna eat something”* (English-speaking female in group 2).

However, parents experienced hardship when their children aged out of WIC benefits, and many expressed difficulties with eligibility for SNAP. Because participants consistently cited WIC, SNAP, and school lunch as most helpful for their children consuming fruits and vegetables, expansions of these programs could alleviate barriers, without creating additional access challenges for families. Participants saw the role of the healthcare institution as a leader in child health policies, and potentially effective advocates for expanding food assistance programs or broader community policies impacting overall household financial stability, and consequently, nutrition and health.

*“…If they [healthcare providers] could advocate for cheaper housing […] then it would be easier to have more food money”* (English-speaking female, Group 2).

*“I feel like [The hospital] is such a huge presence in [the city]. If they were like, ‘There are parents that are not able to get better jobs because they can’t afford the gap between when they get paid and you know the childcare,’ even if they would just like to have some kind of forum where they met with city leaders [and say], ‘This is what we’re hearing from our side of the community and this is what we’re concerned with, being medical people.’”* (English-speaking female, Group 2).

### Increase accessibility

#### Integrate services into “my routine”

Participants recognized the ease of using programs like WIC and SNAP where they could access benefits at times and places that were already “*within the routine […] it wasn’t an extra trip*” (English-speaking female, Group 3).

Participants recalled positive experiences with healthy vending options at hospitals and with programs that were tied to routine activities such as healthcare or grocery store visits that gave free pieces of fruit to children.

#### Communicate programs opportunities often, through multiple channels

Participants felt they would be more likely to access programs if they were publicized in multiple ways and times. Some recalled existing community gardens that were underutilized because people did not know how to get involved. Participants suggested that hospital-based programs (like gardens, educational classes, and support groups) be publicized through their provider during clinic visits, calls to make appointments, appointment reminders, as well as through clinic memo boards, flyers, websites, email and postal mailings.

### Influence desirability

#### Involve children or whole family

Participants emphasized the importance of involving children in programming, particularly in discussions about community gardens, workshops, or rewards programs. In talking about a community garden at the healthcare institution, one participant said,

*“If I got to go and work up there. Ya know, volunteer, then I don’t have to pay for it and I’m actually giving too. If you had kids that could handle something like that, bring your kids, and then they get the opportunity to give back too. And help their self-esteem*” (English-speaking female, Group 1).

About cooking classes, another said,

“*Have the kids in the same group with us, so that way they are interacting too. Because it’s not just us, it’s our kids that have to eat this too, so it should be their opinion too”* (English-speaking female, Group 6).

#### Utilize the unique influence of healthcare providers

In discussing what helped, participants talked about the influence providers can have in promoting FVC. Participants had several programmatic ideas that addressed desirability of fruits and vegetables, many of which involved the healthcare provider as an influencer. For example, fruit baskets at visits and rewards programs would incentivize providers to engage with children, creating positive reinforcement for healthy eating. Healthcare providers could also give handouts with information about healthy cooking or addressing specific nutritional needs.

“*If you tell your doctor, ‘My child isn’t eating right, or he isn’t eating healthy,’ then the doctor will say, ‘Oh look, here is a class we have if you’d like to participate.’”* (Spanish-speaking female, Group 4).

## Discussion

In our qualitative study following an unsuccessful food prescription/mobile market program, caregivers of food-insecure families identified three major interconnected barriers to FVC in their children: affordability, accessibility, and desirability. Participants provided recommendations for healthcare institutions, including family/individual-level programs (e.g. healthy cooking workshops, free fruit/vegetables in clinics), community and organizational-level initiatives (e.g. practical coupons, accessible gardens, linkage to other resources) and policy-level ideas (e.g. hospital-led advocacy for household food and economic policies). Taken together, this patient-centered qualitative evaluation offers critical insight on why previous programs aimed to address FVC may have failed and how future interventions may be more effective.

The barriers to healthy eating identified by our families are similar to previous studies. Most studies have examined low-income populations or have narrowly focused on children diagnosed with chronic health conditions, while our study focused on families with household food insecurity. For food-insecure families, financial constraints are commonly reported barriers [19, 29] centered on the high cost of fruits/vegetables [19] and the lack of resources to purchase them (*affordability)* [18]. Other studies have also identified *accessibility* barriers, including lack of stores selling healthy foods and transportation difficulties [17, 19] to those that do, as well as *desirability* barriers, such as low within-family demand for fruits/vegetables [30], fussy eating habits that limit food exposure practices [31], high time investment to prepare healthy meals [29], and cultural traditions which may emphasize less healthy foods. Using grounded theory methods, our study advances the holistic understanding of barriers among food-insecure families and offers a comprehensive conceptual model to guide future inquiry and program planning.

Although affordability was the most salient barrier discussed in our focus groups, we found that the three main barriers were often interconnected. For example, participants who may not be able to afford food at grocery stores may visit pantries, which were often difficult to access or did not carry fruits and vegetables of a desirable selection or quality. Further, families are concerned about wasting money on vegetables that children may not want to eat or that perish quickly, highlighting the intersection of affordability and desirability.

Other studies have identified intersectionality of barriers, including the low desirability of food options at more accessible locations [32, 33] and the links between affordability and access [17, 29, 34]. The intersection of food insecurity and desirability is further explained in the literature, as mothers of food insecure households were shown to feed their child a narrow range of foods out of concern for food and economic waste, a response that unintentionally limited the child’s exposure to a variety of healthy foods, resulting in pickier eating habits [31].

In examining these three intersecting barriers alongside participant programmatic recommendations, our results highlight the need for hospital-based initiatives to go beyond addressing single barriers and consider affordability, accessibility and desirability factors in their programming.

Our study fills critical gaps in guiding health systems to offer patient-driven, effective programmatic solutions to increase FVC among food-insecure families. Families themselves identified that healthcare institutions can play a distinct role in interventions across the socio-ecological model (Table 2). At the individual/family level, healthcare providers may be able to go beyond providing medical advice and offer fruit and vegetable “prescriptions” or coupons. This concept has limited evidence for effectiveness as only one study of a similar model has shown impact on fruit and vegetable consumption for food-insecure families [16]. This intensive intervention for children with obesity, The Wholesome Wave Fruit and Vegetables Prescription (FVRx) Program, resulted in significant improvements in food security, with greatest improvements among participants with five or more clinic visits over the 6-month study. For a primary care population who are not typically seen with such regularity, the model is not likely to have a large impact on FVC, particularly if delivered in isolation without helping to alleviate related accessibility and desirability barriers. Participants in our pilot program typically received a mobile market coupon during one clinic visit. While one $5 coupon was not likely to alleviate food insecurity nor increase FVC, one aim of the pilot “prescription” was to introduce families to a community resource for low cost fruits and vegetables that serves food deserts in the urban core. This aim was not achieved as the mobile market was not easily accessible for families in the pilot. Additional implementation science research is needed to determine if fruit and vegetable prescription interventions can be adapted to meaningfully address barriers related to food insecurity in primary care populations.

Our participants also recognized that interventions at the individual/family level alone are not likely to be enough. Programs at the community level may be beneficial but need to be designed according to family needs and accompanied by targeted outreach. Participants spoke positively about programs like mobile markets, group classes and community gardens and emphasized them when generating ideas for new programs. However, contrary to wide utilization of programs like WIC, SNAP and school-based food services, very few had reported participating in these existing community-level programs. Low reported utilization of existing community-based programs may be due to access barriers (such as transportation [17] or time constraints [29]), thus, additional effort may be needed to ensure these interventions are compatible enough with participants’ daily realities to become *“part of my routine”.* Participants emphasized involving the child or whole family in many of the program ideas, which may help address barriers around desirability of fruits and vegetables in children and in other family members. As part of a multilevel intervention strategy that includes increasing accessibility and affordability, programs that work to promote desirability at the family-level could help shift preferences of both children and parents, who serve as important role models in their children’s eating behaviors [35, 36]. In particular, multilevel intervention strategies that pay close attention to the impact on specific populations such as families living with food insecurity, can have significant impact on reducing disparities in diet and health [37].

At the organizational level, parents suggested that healthcare providers connect families to community resources, an idea that has also been endorsed by nutrition and research experts [38]. Consistent with existing literature, participants in our study described how their food budgets were limited after paying for “priority” items such as housing, childcare, and medical bills [17]. Healthcare institutions are testing models to employ community navigators or community health workers to connect families to programs for food security and broader services, with promising results [12, 39]. For example, a healthcare system in Colorado implemented community specialists in an active referral process to connect patients with social assistance, including food programs, increasing the percentage of referred patients using a resource hotline from 5% to 75% [12].

On the policy level, our study highlights that patients see health care providers and institutions as advocates for their families for issues from food security to safe affordable housing. Health care institutions’ involvement in policy-level advocacy has been suggested in previous studies that elicit participants’ perspectives on barriers to healthy eating and impacts of food insecurity [40, 41]. In these studies, while participants were not specifically asked about the role of the healthcare institution (as in our study), the authors do conclude that a role exists for the healthcare institution to advocate for policies or programs at multiple levels of the socio ecological model. In our study, participants were asked directly about the role of our healthcare institution related to food insecurity and FVC, and then participants *themselves* made suggestions that included the expanded role for the healthcare institution, such as the policy- level advocacy for issues of food security and broader social determinants of health.

Healthcare institutions and providers could advocate for maintenance and expansion of effective federal and community programs like WIC and SNAP (consistent with American Academy of Pediatricians (AAP) recommendations) [42] as well as for improvement in the nutrition quality of existing emergency food programs. Ensuring access to or expansion of these programs that participants identified as helpful, is a potentially effective strategy in terms of addressing barriers reported.

For example, healthcare institutions could identify strategies to join advocacy efforts connected with professional organizations (i.e. AAP), national anti-hunger organizations or local collaborative groups, where the voice of a healthcare provider or institution could add valuable perspective and knowledge. Healthcare systems with government or community liaisons should work to keep abreast of local and national policy developments, issuing statements or providing testimony in support of expanding food assistance from a healthcare perspective. Considering the range of parent-generated solutions, health care institutions may be able to identify those of which are feasible and which fit into broad strategies being implemented by the institution, or even within the wider community, to address food insecurity and FVC.

This study has several limitations. Its focus on families accessing primary, pediatric care an urban center may not be representative of food-insecure families accessing care in suburban or rural areas, or those who are not connected to formal healthcare services. While males were not excluded from participating in a focus group, only three participants were male, thus, gender influence in perspectives and solutions may not have been fully captured. Solutions generated were solely of the parent perspective and will need to be further explored to determine their feasibility and effectiveness if implemented. Further, while saturation was reached in terms of barriers and facilitators, some new program ideas were generated at each focus group and additional focus groups may have produced additional ideas. Lastly, although participants were encouraged to share both positive and negative experiences, research staff conducting the focus groups were employees of the healthcare center, which may have led to social desirability bias in questions related to the facility’s programs.

## Conclusion

Food insecurity and FVC are complex challenges, affecting both short and long-term health outcomes for children. According to families, healthcare institutions have a role in addressing these challenges on multiple levels. Our study offers a conceptual framework and parent-driven solutions to guide hospitals in defining a strategic, comprehensive role in increasing FVC and its health benefits for children facing household food-insecurity.

## Supplementary information

**Additional file 1.**

## Data Availability

Not applicable.

## Abbreviations

FVC: Fruit and vegetable consumption
SNAP: Supplemental Nutrition Assistance Program
WIC: Women, Infants, and Children
SEEK: Safe Environment for Every Kid
Rx: Prescription

## References

[1] Kim SA, Moore LV, Galuska D, Wright AP, Harris D, Grummer-Strawn LM (2014). Vital signs: fruit and vegetable intake among children - United States, 2003-2010. MMWR Morb Mortal Wkly Rep.

[2] Antova T, Pattenden S, Nikiforov B, Leonardi GS, Boeva B, Fletcher T (2003). Nutrition and respiratory health in children in six central and eastern European countries. Thorax..

[3] Ness AR, Maynard M, Frankel S, Smith GD, Frobisher C, Leary SD (2005). Diet in childhood and adult cardiovascular and all cause mortality: the Boyd Orr cohort. Heart..

[4] Maynard M, Gunnell D, Emmett P, Frankel S, Davey Smith G (2003). Fruit, vegetables, and antioxidants in childhood and risk of adult cancer: the Boyd Orr cohort. J Epidemiol Community Heal.

[5] Shook RP, Halpin K, Carlson JA, Davis A, Dean K, Papa A (2018). Adherence with multiple National Healthy Lifestyle Recommendations in a large Pediatric Center electronic health record and reduced risk of obesity. Mayo Clin Proc.

[6] Coleman-Jensen A, Rabbitt MP, Gregory C, Singh A (2016). Household food security in the United States in 2014. US Household Food Security: Statistics and Analysis for 2014.

[7] Coleman-Jensen A, Gregory C, Singh A (2015). Household food security in the United States in 2013.

[8] Bruening M, MacLehose R, Loth K, Story M, Neumark-Sztainer D (2012). Feeding a family in a recession: food insecurity among Minnesota parents. Am J Public Health.

[9] Kendall A, Olson CM, Frongillo EA (1996). Relationship of hunger and food insecurity to food availability and consumption. J Am Diet Assoc.

[10] Support S. Social Determinants of Health Series: food insecurity and the role of hospitals food insecurity and the role of hospitals social determinants of health series 2 social determinants of health series: food insecurity and the role of hospitals. Suggest Cit Heal Res Educ Trust. 2017; Available from: https://www.aha.org/system/files/hpoe/Reports-HPOE/2017/determinants-health-food-insecurity-role-of-hospitals.pdf.

[11] Palakshappa D, Doupnik S, Vasan A, Khan S, Seifu L, Feudtner C (2017). Suburban families’ experience with food insecurity screening in primary care practices. Pediatrics..

[12] Exploratory Evaluation of Food Insecurity Programs Initiated by Health Care Organizations. San Francisco; 2016. Available from: https://nopren.org/wp-content/uploads/2017/01/TH-Exploratory-Evaluation-Summary-FINAL1.pdf.

[13] Burkhardt MC, Beck AF, Conway PH, Kahn RS, Klein MD (2012). Enhancing accurate identification of food insecurity using quality-improvement techniques. Pediatrics..

[14] Adams E, Hargunani D, Hoffmann L, Blaschke G, Helm J, Koehler A (2017). Screening for food insecurity in Pediatric primary care: a Clinic’s positive implementation experiences. J Health Care Poor Underserved.

[15] Bottino CJ, Rhodes ET, Kreatsoulas C, Cox JE, Fleegler EW (2017). Food insecurity screening in Pediatric primary care: can offering referrals help identify families in need?. Acad Pediatr.

[16] Ridberg RA, Bell JF, Merritt KE, Harris DM, Young HM, Tancredi DJ (2018). A Pediatric Fruit and Vegetable prescription program increases food security in low-income households. J Nutr Educ Behav.

[17] Demartini TL, Beck AF, Kahn RS, Klein MD (2013). Food insecure families: description of access and barriers to food from one pediatric primary care center. J Community Health.

[18] Edin K, Boyd M, Mabli J, Ohls J, Worthington J, Greene S (2013). SNAP Food Security In-Depth Interview Study. U.S. Department of Agriculture, Food and Nutrition Service, Office of Research and Analysis.

[19] Dave JM, Thompson DI, Svendsen-Sanchez A, Cullen KW (2017). Perspectives on barriers to eating healthy among food pantry clients. Heal Equity.

[20] The State of the Children’s Health. 2016 Community health needs assessment for the Kansas City region. Kansas City; 2016. Available from: https://www.childrensmercy.org/siteassets/media-documents-for-depts-section/documents-for-in-the-community/community-benefit/community-health-needs-assessment-2016.pdf.

[21] Lane W, Dubowitz H, Feigelman S, Poole G (2014). The effectiveness of food insecurity screening in Pediatric primary care. Int J Child Heal Nutr.

[22] Saldaña J (2015). The coding manual for qualitative researchers. Sage.

[23] Kruger RA. Moderating focus groups, vol. 4. Thousand Oaks: Sage Publications; 1997.

[24] Dedoose. Los Angeles, CA: SocioCultural Research Consultants, LLC; Available from: https://app.dedoose.com/App/?Version=8.2.14. Accessed 7 Aug 2018.

[25] Charmaz K. Constructing Grounded Theory: A Practical Guide through Qualitative Analysis., 2nd Ed. Thousand Oaks: Sage Publications; 2014.

[26] Story M, Kaphingst KM, Robinson-O’Brien R, Glanz K (2008). Creating healthy food and eating environments: policy and environmental approaches. Annu Rev Public Health.

[27] Paskett E, Thompson B, Ammerman AS, Ortega AN, Marsteller J, Richardson DJ (2016). Multilevel interventions to address health disparities show promise in improving population health. Health Aff.

[28] Golden SD, Earp JAL (2012). Social ecological approaches to individuals and their contexts: twenty years of Health Education & Behavior Health Promotion Interventions. Heal Educ Behav.

[29] Mook K, Laraia BA, Oddo VM, Jones-Smith JC (2016). Food security status and barriers to Fruit and vegetable consumption in two economically deprived communities of Oakland, California, 2013–2014. Prev Chronic Dis.

[30] Baranowski T, Baranowski J, Cullen KW, DeMoor C, Rittenberry LT, Hebert D (2002). 5 a day achievement badge for African-American boy scouts: pilot outcome results. Prev Med (Baltim).

[31] Harris HA, Staton S, Morawska A, Gallegos D, Oakes C, Thorpe K (2019). A comparison of maternal feeding responses to child fussy eating in low-income food secure and food insecure households. Appetite..

[32] Gittelsohn J, Rowan M, Gadhoke P (2012). Interventions in small food stores to change the food environment, improve diet, and reduce risk of chronic disease. Prev Chronic Dis.

[33] Simmet A, Depa J, Tinnemann P, Stroebele-Benschop N (2017). The nutritional quality of food provided from food pantries: a systematic review of existing literature. J Acad Nutr Diet.

[34] Evans A, Banks K, Jennings R, Nehme E, Nemec C, Sharma S (2015). Increasing access to healthful foods: a qualitative study with residents of low-income communities. Int J Behav Nutr Phys Act.

[35] Pearson N, Biddle SJH, Gorely T (2009). Family correlates of fruit and vegetable consumption in children and adolescents: a systematic review. Public Health Nutr.

[36] Knai C, Pomerleau J, Lock K, McKee M (2006). Getting children to eat more fruit and vegetables: a systematic review. Prev Med (Baltim)..

[37] Peeters A, Blake MRC (2016). Socioeconomic inequalities in diet quality: from identifying the problem to implementing solutions. Curr Nutr Rep.

[38] Kirkpatrick SI (2012). Understanding and addressing barriers to healthy eating among low-income Americans. J Acad Nutr Diet.

[39] Stenmark S. Lessons learned from implementation of the food insecurity screening and referral program at Kaiser Permanente Colorado. Perm J. 2018:58–64. 10.7812/TPP/18-093 PMC6175601 30296400

[40] Knowles M, Rabinowich J, Ettinger de Cuba S, Cutts DB, Chilton M (2016). “Do you Wanna breathe or eat?”: parent perspectives on child health consequences of food insecurity, trade-offs, and toxic stress. Matern Child Health J.

[41] Haynes-Maslow L, Auvergne L, Mark B, Ammerman A, Weiner BJ (2015). Low-income individuals’ perceptions about fruit and vegetable access programs: a qualitative study. J Nutr Educ Behav.

[42] NUTRITION COCP and CO (2015). Promoting food security for all children. Pediatrics.

