# Supplementary material for: Beyond clinical food prescriptions and mobile markets: parent views on the role of a healthcare institution in increasing healthy eating in food insecure families
Source: Nutr J. 2020 Sep 9;19:94. doi: 10.1186/s12937-020-00616-x (PMC7487727; doi:10.1186/s12937-020-00616-x)
Supplement: Supplementary file 1 — Additional file 1. [file 12937_2020_616_MOESM1_ESM.docx]

|  | **Question** | **Goal of Question** | **Time** |
| --- | --- | --- | --- |
|  | Icebreaker (at end of intro): **Introduce yourself and tell me about one of your family’s favorite meals?** | Break the Ice, everyone shares something | 3 |
| 1. | **Who would want to share about the fruits and vegetables that your kids like to eat.” Or share about the fruits and vegetables they hate to eat!”** | Ease into thinking about/talking about food and fruits and veggies; still break the ice and start the pattern of conversation | 5 |
| 2 | **In thinking about food, what are your biggest worries when it comes to food and your family?**  *If no answers, “In your community, your neighbors or friends, what do you think are the biggest worries related to food?”*    **What would you say is most important in helping in times when food or food concerns are a major worry?** | Understand what they are concerned about related to food broadly,  Feel that their concerns are heard related to food and/or healthy food  Identify/start thinking about current strategies | 10 |
| 3 | *Listen for mention of healthy food…* ***if*** *healthy food is mentioned:*  **It sounds like [repeat what was heard above related to healthy food] getting enough healthy food is one worry. When you talk about healthy foods, what kinds of foods do you think of?**  *Listen for mention of fruits and veggies…* ***if*** *fruits and veggies are mentioned:*  **Someone mentioned fruits and vegetables, specifically. That is one type of healthy food that we’ve heard from other families about.**  **What are the reasons you think families in our community do not eat more fruits and vegetables?**  What do you think makes it hard for families to eat fruits and vegetables?  Is there anything else that hasn’t been mentioned that makes it hard for your family to get more fruits and veggies?  *Additional Probe if needed:* Think about your family’s favorite meal you said in the beginning. How possible would it be add a vegetable to this? What would you add? What might be some of the challenges you would face in doing this?  **What are the reasons you think families in our community do eat fruits and vegetables?**  What makes it easy to eat fruits and vegetables?  Is there anything else that hasn’t been mentioned that makes it easier for your family to get more fruits and veggies?  *Additional Probe if needed, “What are the tricks and tips that you use to get around some of these things that make it hard?”* | Understand definition of healthy, according to them  Narrow conversation to healthy and then to fruits and vegetables  Identify barriers to getting fruits and veggies  Identify things that help in getting fruits and veggies | 10 |
| 4 | **So we’ve talked about worries around food, about healthy food -specifically fruits and vegetables, and some of the challenges and things that make it easier.**  **One of the reasons we are having these discussions is because the CMH doctors' know that having enough food and having enough healthy food can really impact a kid’s health. They are interested in your opinions and thoughts about things that a healthcare provider might do that would help families get the healthy foods they need.**  **A program that CMH tried last year was a “food prescription”. The healthcare providers gave families a “food prescription” – which included a $5 coupon for a mobile market that sells low cost fresh fruits and veggies.**  **Here’s some basic info about the program (flip chart of who, what when where why- read through).**  **Most families didn’t use the $5 coupon, and we know that many people said barriers were that they forgot about the coupon, they were too busy, or that it was difficult to go to the mobile market- like the times and locations made it hard.**  **But CMH still has coupons left to use up, and so will be starting to give out the food prescriptions again this month.**  **Are there any changes that could be made, so that this would work better for your family?**  *Probes if needed: What would be the best way to make this work for you?*    HALF WAY POINT | Get ideas on what can tweak right now on Food Rx  Show them the who, what, when, where, why idea for the food rx to prep for the next question  *Don’t spend much too time- more interested in next question than this one.* | 10 |
| 5 | **Start by 6:45**  **Now, thinking about these same issues and what CMH might do to help families eat more fruits and veggies.**  **Imagine you had a clean slate and you were in charge of creating your own program from scratch. You were wanting to do something to make it easier for families to eat healthy foods, and Children’s Mercy would be involved. What would you do? What would your program look like?**  **Any ideas or thoughts are welcome- don’t feel like it has to be a full program.**  **Pause here- as an idea starts to take shape, possible begin filling out flip chart, probing about the who, what, when, where why….**  **We’ll take notes as you are talking here- and even start to fill in the who, what, when, where and why- like we did on the food prescription.**  **Start by 7:00**  *Probes:*  *What kind of things could Children’s Mercy do?*  *What kinds of thing would be provided? Probe: Food itself, coupons, resources, connection to a program*  *What location?*  *What time?*  *Who would be able to use the program? (Who would the program be for?)*  *How would you want to hear about this program?*  *What would be the biggest benefit?*  *Would there be any barriers?*  *Moderator makes a list on the flip chart of ideas generated by the group*  *If we need prompts*, *some ideas we’ve heard have been coupons for fruits and veggies, cooking groups, having a food pantry or providing food onsite at clinic appointments, free lunch onsite for children, having a community garden nearby, having WIC onsite, and providing a list of food pantries that have fruits and veggies.* | Brainstorm ideas for a program or service  Understand characteristics of the program  Understand how they see the role of CMH/healthcare if at all  Get more specific details about how a program might work, what is most important to them  *Model with Food Rx to help it be more concrete* | 25 |
| 6*.* | **Now, we’d like to think about all of these ideas here that we discussed. Out of this list think about which one or two might be most helpful to you, or to other families that you know.**  **Why do you all think this idea rose to the top?**  *Probe: what do you like best about this idea? What would be most important for CMH to think about related to this idea?* | Ranking to understand their priority  Most importantly, understanding why /hearing more about what is important to them  See if food Rx 2.0 is an idea they like in comparison to others | 10 |
| 7. | (ask assistant moderator summarizes the discussion for the group- mainly the last two questions) **Was there anything I missed?** |  | 3 |
| 8. | **Is there anything else you want us to know?** |  | 3 |
